# Improved recovery of regional left ventricular function after PCI of chronic total occlusion in STEMI patients: a cardiovascular magnetic resonance study of the randomized controlled EXPLORE trial

**DOI:** 10.1186/s12968-017-0369-z

**Published:** 2017-07-19

**Authors:** Joëlle Elias, Ivo M. van Dongen, Loes P. Hoebers, Dagmar M. Ouweneel, Bimmer E. P. M. Claessen, Truls Råmunddal, Peep Laanmets, Erlend Eriksen, René J. van der Schaaf, Dan Ioanes, Robin Nijveldt, Jan G. Tijssen, Alexander Hirsch, José P. S. Henriques, on behalf of the EXPLORE investigators

**Affiliations:** 1 Academic Medical Center - University of Amsterdam, Amsterdam, the Netherlands; 2 Sahlgrenska University Hospital, Gothenburg, Sweden; 3 North Estonia Medical Center, Tallinn, Estonia; 4 Haukeland University Hospital, Bergen, Norway; 5 Onze Lieve Vrouwe Gasthuis, Amsterdam, the Netherlands; 6 VU Medical Center, Amsterdam, the Netherlands; 7 Erasmus Medical Center, Rotterdam, the Netherlands; 8 Department of Cardiology, Academic Medical Center - University of Amsterdam, Meibergdreef 9, 1105 AZ Amsterdam, The Netherlands

**Keywords:** CTO, STEMI, PCI, LV function

## Abstract

**Background:**

The Evaluating Xience and left ventricular function in PCI on occlusiOns afteR STEMI (EXPLORE) trial did not show a significant benefit of percutaneous coronary intervention (PCI) of the concurrent chronic total occlusion (CTO) in ST-segment elevation myocardial infarction (STEMI) patients on global left ventricular (LV) systolic function. However a possible treatment effect will be most pronounced in the CTO territory. Therefore, we aimed to study the effect of CTO PCI compared to no-CTO PCI on the recovery of regional LV function, particularly in the CTO territory.

**Methods:**

Using cardiovascular magnetic resonance (CMR) we studied 180 of the 302 EXPLORE patients with serial CMR (baseline and 4 months follow-up). Segmental wall thickening (SWT) was quantified on cine images by an independent core laboratory. Dysfunctional segments were defined as SWT < 45%. Dysfunctional segments were further analyzed by viability (transmural extent of infarction (TEI) ≤50%.). All outcomes were stratified for randomization treatment.

**Results:**

In the dysfunctional segments in the CTO territory recovery of SWT was better after CTO PCI compared to no-CTO PCI (ΔSWT 17 ± 27% vs 11 ± 23%, *p* = 0.03). This recovery was most pronounced in the dysfunctional but viable segments(TEI < 50%) (ΔSWT 17 ± 27% vs 11 ± 22%, *p* = 0.02). Furthermore in the CTO territory, recovery of SWT was significantly better in the dysfunctional segments in patients with Rentrop grade 2–3 collaterals compared to grade 0–1 collaterals to the CTO (16 ± 26% versus 11 ± 24%, *p* = 0.04).

**Conclusion:**

CTO PCI compared with no-CTO PCI is associated with a greater recovery of regional systolic function in the CTO territory, especially in the dysfunctional but viable segments. Further research is needed to evaluate the use of CMR in selecting post-STEMI patients for CTO PCI and the effect of regional LV function recovery on clinical outcome.

**Trial registration:**

Trialregister.nl NTR1108, Date registered NTR: 30-okt-2007.

## Background

ST-segment elevation myocardial infarction (STEMI) patients with a concurrent chronic total occlusion (CTO), found in 10–15% of STEMI patients, have a remarkably higher mortality and morbidity [1, 2]. In STEMI patients with a concurrent CTO, the Evaluating Xience and left ventricular function in PCI on occlusiOns afteR STEMI (EXPLORE) was the first randomized trial that compared CTO percutaneous coronary intervention (PCI) within one week after STEMI versus no-CTO PCI within 4 months. This study showed that CTO PCI compared to no-CTO PCI did *not* result in a higher left ventricular (LV) ejection fraction (EF) and lower left ventricular end-diastolic volume (LVEDV) measured by cardiovascular magnetic resonance (CMR) at 4 months follow-up (FU) [3]. An in-depth analysis on the recovery of global LV function (comparing LV function at baseline to 4 month) in 180 patients with serial CMR showed that there was no treatment effect of CTO PCI[4]. The primary endpoints of EXPLORE were global LVEF and LVEDV which are parameters that are affected by many other factors, especially in the early post-STEMI phase. A possible treatment effect of CTO PCI will however be most pronounced in the CTO territory. We therefore aimed to perform an in-depth quantitative analysis of the regional function of the myocardium supplied by the CTO. CMR allows for accurate analysis of regional segmental function (segmental wall thickening (SWT)) [5, 6]. In elective setting, successful CTO PCI has been associated with significant improved regional wall motion over time, especially in dysfunctional but viable segments (transmural extent of infarction (TEI) <50%) [6, 7]. However the effect of CTO PCI on regional function has never been compared to a control group (no-CTO PCI) and it has not been investigated in STEMI patients with a concurrent CTO. Therefore in this EXPLORE CMR study we aimed to study the effect of CTO PCI compared to no-CTO PCI on the recovery of regional LV function, particularly in the CTO territory.

## Methods

### Patients and treatment

The design and main results of the EXPLORE trial were described in detail previously [3]. Briefly, the EXPLORE study was a randomized multi-centre clinical trial in which STEMI patients with a concurrent CTO (*n* = 302) between 2007 and 2015 were randomly assigned in a 1:1 ratio to CTO PCI within 7 days after primary PCI (*n* = 148) or to a conservative strategy (no-CTO PCI) for at least 4 months (*n* = 154). Patients were eligible if during successful primary PCI a concurrent CTO was found in a non-infarct related artery (IRA). Important exclusion criteria were >48 h hemodynamically instability and conditions impeding CMR imaging such as renal failure, atrial fibrillation and indication for pacemaker or implantable cardioverter-defibrillator (ICD) within 4 months after randomization. The primary endpoints of the trial were LVEF and LVEDV at 4 months follow-up measured on CMR. At 4 months follow-up there was no significant difference on LV function between patients randomized to CTO PCI and patients randomized to no-CTO PCI (LVEF: 44.1 ± 12.2% vs. 44.8 ± 11.9%, *p* = 0.60 and LVEDV 215.6 ± 62.5 ml versus 212.8 ± 60.3 ml, *p* = 0.70). Also, no significant differences in the secondary endpoints of left ventricular systolic volume, left ventricular mass and infarct size were observed. CTO was defined as a 100% luminal narrowing without antegrade flow or with antegrade or retrograde filling through collaterals. The coronary vessel in which the CTO was located should have a reference diameter of at least 2.5 mm. For patients with multiple CTOs, the main CTO was defined as the CTO supplying the largest amount of myocardium. All coronary angiographies were evaluated by an independent angiography corelab to assess location and characteristics of culprit lesion, CTO lesions and quality of the collaterals. Collateral quality assessment was done at the baseline angiogram (primary PCI). Presence and extent of retrograde collateral flow was determined using the Rentrop classification score [8]. Patients were divided into 2 groups: patients with angiographic grade 0 or 1 collaterals to the CTO and patients with angiographic grade 2 or 3 collaterals to the CTO territory. If a patient had ≥1 collateral to the CTO, the collateral with the highest score was used. The current study cohort includes 180 of the 302 included EXPLORE patients and is a substudy performed in patients with serial CMR (baseline and 4 months FU) and this cohort has been described before [4]. The primary outcome of this EXPLORE substudy is recovery of regional LV function (SWT %) from baseline to 4 months follow-up defined as change in SWT, specifically measured in the CTO supplied territory. Furthermore we assessed the effect of CTO PCI in the dysfunctional but viable segments and the effect of collaterals on regional LV function.

### Cardiovascular magnetic resonance protocol

CMR was performed on a 1.5-Tesla scanner using a dedicated phased array cardiac receiver coil. For LV function imaging, during repeated breath holds, ECG-gated balanced steady-state free-precession cine images were obtained in short-axis orientation covering the left ventricle from base to apex. For infarct analysis of the myocardium, at least 10 min after administration of a gadolinium-based contrast agent, late gadolinium-enhanced (LGE) images were acquired using an inversion recovery gradient-echo pulse sequence with slice locations identical to the cine images.

### Segmental analysis

A 16-segment model, excluding the apex, was used to analyze the segmental function in each patient. Endo- and epicardial borders were manually outlined on all short-axis cine slides on the end-diastolic and end-systolic images, which has been shown to have a high reproducibility and reliability [9, 10]. SWT was defined as a percentage increase of LV wall thickness during systole compared with diastole [11]. Myocardial segments were considered dysfunctional if SWT was less than 45% [12]. TEI was used to assess viability, which was calculated by dividing the hyper enhanced area by the total area in each of the 16 segments and expressed as a percentage [13]. The TEI was divided into 2 groups: 0%–50% and >50% per segment. Four month follow-up CMR was made according to the same protocol. For each patient individual segments were assigned to one of the major coronary arteries using the American Heart Association standardized myocardial segmentation and nomenclature statement for tomographic imaging of the heart. Using this standard model we determined whether segments were supplied by the CTO, IRA or remote (related to the baseline coronary anatomy scored by the angio corelab) [14]. We studied the effect of CTO PCI on recovery of regional SWT. To study changes over time baseline and 4 month CMR were compared. To study the effect of revascularization on regional function, segments in the perfusion territory of the CTO and IRA were analyzed separately. All CMR images were analysed by an independent core laboratory, blinded for randomization outcome, (ClinFact Corelab, Leiden) using dedicated software (QMass MR analytical software version 7.6, Medis BV, Leiden, the Netherlands). Quality control of the CMR data was performed by one person (RN) and in case of poor imaging quality or artefacts hampering imaging analysis, the CMR data were excluded.

### Statistical analysis

Data are presented as mean ± standard deviation for continuous variables. Discrete variables were summarized as frequencies and percentages. Baseline characteristics were compared using the independent-samples T-test, or Fisher’s exact probability test in case of binary endpoints. Changes in LVEF, LVEDV and infarct size within each group were tested with paired student t-test. We evaluated the recovery of regional segmental outcome (percentage of SWT) in relation to the presence of dysfunctional segments at baseline and the presence of viability (TEI <50%) at baseline. All outcomes were stratified for randomization treatment. Because within 1 patient the regional function in the different segments is strongly related and not an independent outcome, multilevel analysis was used (linear regression) [11]. In the recovery of regional function analysis, a correction for baseline percentage of SWT was made. All tests were 2 sided, and a *p*-value <0.05 was considered to indicate statistical significance.

## Results

### Baseline clinical and procedural characteristics

**Fig. 1:**
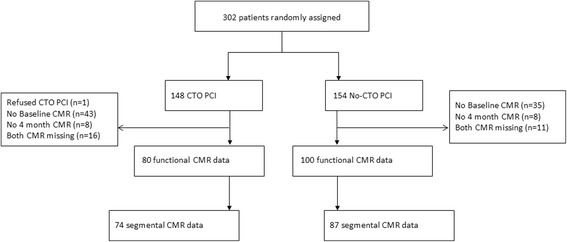
Flowchart included patients. CMR = cardiovascular magnetic resonance, CTO = chronic total occlusion, PCI = percutaneous coronary intervention

From the 302 patients randomized in the EXPLORE Trial, 180 patients completed serial CMR (baseline and 4 months FU) and were included in the present analysis (Fig. 1). The baseline characteristics of the 180 patients included in this study and all patients in the EXPLORE study are shown in Table 1. Sixty percent of the patients in the CTO PCI group and 53% of the patients in the no-CTO PCI had Rentrop grade 2–3 collaterals to the CTO territory. Baseline characteristics were comparable between the patients with serial CMR and patients without serial CMR (Table 1). In 161 of the 180 patients CMR quality was sufficient to assess regional function, regional CMR data from 19 patients were excluded because of insufficient imaging quality.

**Table 1:** Baseline characteristics of STEMI patients with a CTO, stratified for the randomization outcome, in total group and in patients with serial CMR

	Serial CMR (*n* = 180)		Total (*n* = 302)		
	CTO-PCI (*n* = 80)	No CTO-PCI (*n* = 100)	CTO-PCI (*n* = 148)	No CTO-PCI (*n* = 154)	*P*-value*
Age (years, mean, SD)	60(10)	60(10)	60(10)	60(10)	0.81
Male gender (%)	73(91)	84(84)	131(89)	126(82)	0.25
Diabetes (%)	13(16)	14(14)	22(15)	25(16)	0.75
Hypertension (%)	30(38)	48(48)	59(40)	69(45)	0.72
Family history of CAD (%)	32(40)	38(38)	66(45)	64(42)	0.10
Hypercholesterolaemia (%)	27(34)	33(33)	51(35)	52(34)	0.81
Current smoker (%)	40(50)	52(52)	77(52)	76(49)	0.97
Previous MI (%)	9(11)	17(17)	19(13)	24(16)	1.0
Previous PCI (%)	5(6)	13(13)	9(6)	16(10)	0.21
Previous stroke (%)	2(3)	3(3)	5(3)	6(4)	0.36
Primary PCI					
Infarct related artery					0.77
Right coronary artery (%)	26(32)	31(31)	46(31)	47(31)	
Left circumflex artery (%)	15(19)	30(30)	30(20)	43(28)	
Left anterior descending artery/LM (%)	39(49)	39(39)	72(49)	64(42)	
Three vessel disease (%)	34(43)	42(42)	62(42)	67(44)	0.91
MI SYNTAX score I (pre-PCI)	30(8)	29(10)	29(8)	29(10)	0.57
MI SYNTAX score II (wiring/balloon/aspiration)	28(8)	26(10)	27(8)	27(10)	0.62
Infarct size					
Peak CK-MB (median, IQR)	133(38–198)	87(39–199)	130(39–272)	111(43–256)	0.14
Peak Troponin T(median, IQR)	3.1(1.3–7.8)	2.9(0.9–5.6)	3.1(1.1–7.8)	3.3(0.9–6.0)	0.23
LVEF before randomization ^a^	41(12)	42(12)	41(11)	42(12)	0.97
CTO characteristics					
Patients with multiple CTOs ^b^	11(14)	13(13)	13(9)	22(14)	0.28
CTO related artery					0.61
Right coronary artery	32(40)	54(54)	46(43)	78(51)	
Left circumflex artery	23(29)	24(24)	48(32)	37(24)	
Left anterior descending artery	25(31)	22(22)	36(24)	39(25)	
CTO Collaterals Rentrop Grade 2–3	48(60)	53(53)	80(54)	82(53)	0.30
Total J-CTO score (mean, SD)	2.0(1.1)	2.2(1.1)	2.1(1.1)	2.3(1.1)	0.07
Previously failed lesion	1(1)	4(4)	2(1)	4(3)	0.41
Blunt stump	17(21)	27(27)	33(22)	45(29)	0.51
Bending	50(63)	68(68)	98(66)	108(70)	0.26
Calcification	58(73)	81(81)	115(78)	132(86)	0.02
Occlusion length ≥ 20 mm	30(38)	43(43)	60(40)	68(44)	0.48

Data are number of patients (%), mean (SD) or median (IQR)

*PCI* percutaneous coronary intervention, *CK-MB* creatine kinase-MB isoenzyme, *CTO* chronic total occlusion, *J-CTO* Multicenter CTO registry of Japan, *CMR* cardiovascular magnetic resonance

*p-value for patients with serial CMR versus patients without serial CMR

^a^Imaging modality is CMR only; data available in 201 patients

^b^For patients with multiple CTOs, the CTO supplying the largest amount of myocardium was defined as the main CTO

### Recovery of global left ventricular function

Recovery of global functional outcome was assessed in 180 patients with serial CMR. Compared to baseline, at 4 months LVEF increased with 4.6 ± 8.3% in patients undergoing CTO PCI versus 3.8 ± 8.1% in the no-CTO PCI group (*p* = 0.52), for LVEDV it was 5.5 ± 32.2 ml versus 3.0 ± 25.7 ml (*p* = 0.57). There was no significant difference in functional recovery between CTO PCI and no-CTO PCI, also the decrease in infarct size was not different between the 2 groups (Table 2).

**Table 2:** Serial CMR outcomes: Recovery of global functional outcomes in the total CMR population

Total CMR population	CTO PCI (*n* = 80)	No-CTO PCI (*n* = 100)	*P*-value
Left ventricular ejection fraction (%)			
Baseline	40.6 (11.8)	41.7 (12.1)	0.55
4 months FU	45.3 (11.6)	45.5 (11.8)	0.87
Δ LVEF	4.6 (8.3)	3.8 (8.1)	0.52
Left ventricular end-diastolic volume (ml)			
Baseline	210.1 (53.4)	209.5 (55.1)	0.95
4 months FU	215.6 (54.6)	212.5 (53.9)	0.71
Δ LVEDV	5.5 (32.4)	3.0 (25.7)	0.57
Infarct size (g) ^a^			
Baseline	11.7 (10.5)	11.9(11.1)	0.94
4 months FU	7.5 (6.4)	7.1 (5.4)	0.70
Δ Infarct size	−4.3 (8.0)	−4.8 (8.6)	0.72

Data are number of patients (n)

*CMR* cardiac magnetic resonance imaging, *CTO* chronic total occlusion, *PCI* percutaneous coronary intervention, *LAD* left anterior descending artery, *LVEF* left ventricular ejection fraction, *LVEDV* left ventricular end-diastolic volume

^a^Infarct data available in 60 and 68 patients

### Recovery of overall segmental wall thickening

A total of 2576 segments (*n* = 161 patients) were available for analysis of function (percentage SWT at 4 month FU compared to baseline). Although a significant recovery of SWT was seen over time, there was no difference between the treatment arms (CTO PCI: 7 ± 30% versus no-CTO PCI: 4 ± 30%, p = NS). In the segments that were dysfunctional at baseline (*n* = 1511) no significant recovery of SWT was seen in the CTO PCI group compared to no-CTO PCI (16 ± 26% versus 13 ± 24% respectively, *p* = 0.06). In dysfunctional segments with TEI < 50% (*n* = 1127) there was also no significant difference on the recovery of SWT after CTO PCI compared to no-CTO PCI (16 ± 26% versus 13 ± 24%, *p* = 0.06). In dysfunctional segments with TEI > 50% (*n* = 82) there was no significant difference in recovery of SWT between CTO PCI and no-CTO PCI (11 ± 17% versus 12 ± 24%, p = NS). Table 3 shows the change in percentage SWT in all segments, in dysfunctional segments and in dysfunctional segments with transmural extent of infarction (TEI) < 50% and TEI >50% comparing CTO PCI versus no-CTO PCI.

**Table 3:** Serial CMR outcomes: Recovery of regional segmental outcomes (SWT), in the total CMR population and according to the territory at risk

Segmental Wall thickening (%, SD)	Total			CTO territory			IRA territory		
All segments	CTO PCI (s = 1184)	No-CTO PCI (s = 1392)	*P*-value*	CTO-PCI (s = 392)	No-CTO PCI (*n* = 453)	*p*-value*	CTO PCI (s = 397)	No-CTO PCI (s = 471)	*p*-value*
Baseline	41 (34)	43 (34)	–	44 (35)	40 (33)	–	31 (28)	33 (31)	–
4 mo	48 (32)	46 (33)	–	49 (33)	43 (31)	–	42 (32)	42 (34)	–
Absolute difference	7 (30)	4 (30)	–	5 (31)	3 (28)	0.09	11 (28)	8 (30)	–
Dysfunctional segments (SWT Baseline < 45%)	CTO PCI (s = 722)	No-CTO PCI (s = 789)	*p*-value*	CTO PCI (s = 226)	No-CTO PCI (s = 275)	*p*-value*	CTO PCI (s = 297)	No-CTO PCI (s = 316)	*p*-value*
Baseline	20 (15)	19 (16)	–	21 (16)	19 (16)	–	18 (15)	16 (17)	–
4 mo	36 (28)	31 (25)	0.05	37 (30)	30 (24)	0.02	33 (27)	30 (26)	–
Absolute difference	16 (26)	13 (24)	0.06	17 (27)	11 (23)	0.03	16 (24)	14 (25)	–
Dysfunctional and TEI <50%	CTO PCI (s = 574)	No-CTO PCI (s = 553)	*p*-value*	CTO PCI (*n* = 186)	No-CTO PCI (*n* = 202)	*p*-value*	CTO PCI (s = 222)	No-CTO PCI (s = 206)	*p*-value*
Baseline	21 (15)	20 (16)	–	22 (16)	20 (16)	–	20 (14)	18 (16)	–
4 mo	38 (28)	33 (25)	0.05	40 (30)	31 (23)	0.01	37 (27)	33 (26)	–
Absolute difference	16 (26)	13 (24)	0.06	17 (27)	11 (22)	0.02	17 (25)	15 (25)	–
Dysfunctional and TEI >50%	CTO PCI (s = 36)	No-CTO PCI (s = 46)	*p*-value*	CTO-PCI (s = 8)	No-CTO PCI (s = 7)	*p*-value*	CTO PCI (s = 27)	No-CTO PCI (s = 37)	*p*-value*
Baseline	8 (12)	3 (15)	–	3 (14)	7 (13)	–	9 (12)	2 (16)	–
4 mo	18 (17)	15 (21)	–	20 (15)	10 (15)	–	18 (18)	14 (21)	–
Absolute difference	11 (17)	12 (24)	–	16 (20)	3 (18)	–	9 (16)	12 (24)	–

Data are percentage of segmental wall thickening (± SD)

*S* number of segments, *CMR* cardiovascular magnetic resonance, *CTO* chronic total occlusion, *PCI* percutaneous coronary intervention, *IRA* infarct-related artery, *SWT* segmental wall thickening, *TEI* transmural extend of infarction

*Outcomes were analyzed using multilevel analysis (linear regression), only *p*-values ≤0.10 are given

### Recovery of regional segmental function of the CTO territory

**Fig. 2:**
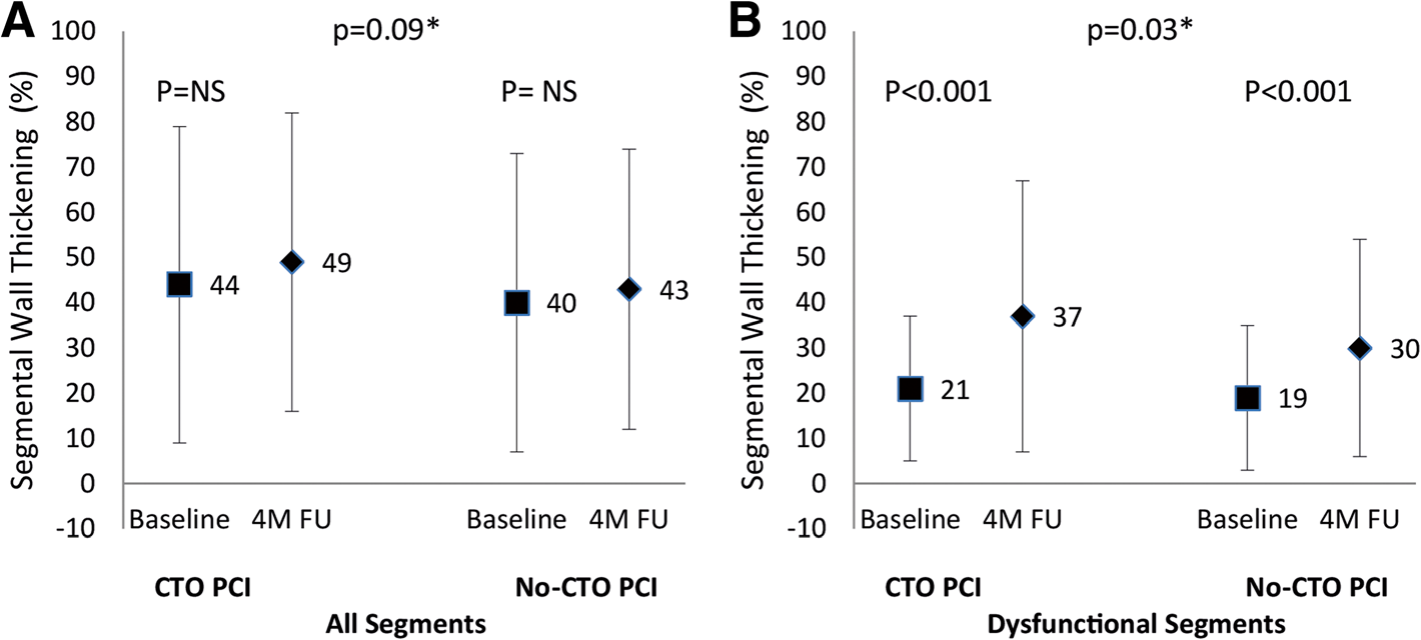
Changes in regional left ventricular function in segments in the CTO territory, comparing CTO PCI versus no-CTO PCI. Change in percentage segmental wall thickening (SWT) in all segments and dysfunctional segments in the CTO territory (**a** and **b**). Recovery of SWT was compared between baseline and 4 month follow-up in segments and between CTO PCI and no-CTO PCI (*)

There were 845 segments in the CTO territory available for analysis (Table 3). In CTO territory segments recovery of SWT was not significantly better in the CTO PCI group compared to no-CTO PCI. In dysfunctional segments, the SWT recovery was most noticeable: PCI of the CTO compared to a conservative approach resulted in a significantly better recovery of SWT (17 ± 27% vs 11 ± 23%, *p* = 0.03). In dysfunctional segments with TEI <50% in the CTO territory CTO PCI resulted in a significantly better recovery of SWT compared to no-CTO PCI (17 ± 27% versus 11 ± 22%, *p* = 0.02). In dysfunctional segments with TEI > 50% in the CTO territory there was no significant difference in recovery after CTO PCI compared to no-CTO PCI (16 ± 20% versus 3 ± 18%, *p* = NS). Figure 2 shows the recovery of SWT in the CTO territory according to the treatment arms.

### Recovery of regional segmental function of the IRA territory

There were 868 segments in the IRA territory available for analysis. The highest recovery was seen in the segments in the IRA territory, however CTO PCI did *not* lead to a better improvement of SWT. In dysfunctional segments in the IRA territory the recovery was comparable between the treatment groups (Table 3).

### Influence of collaterals to the CTO territory on regional function

**Fig. 3:**
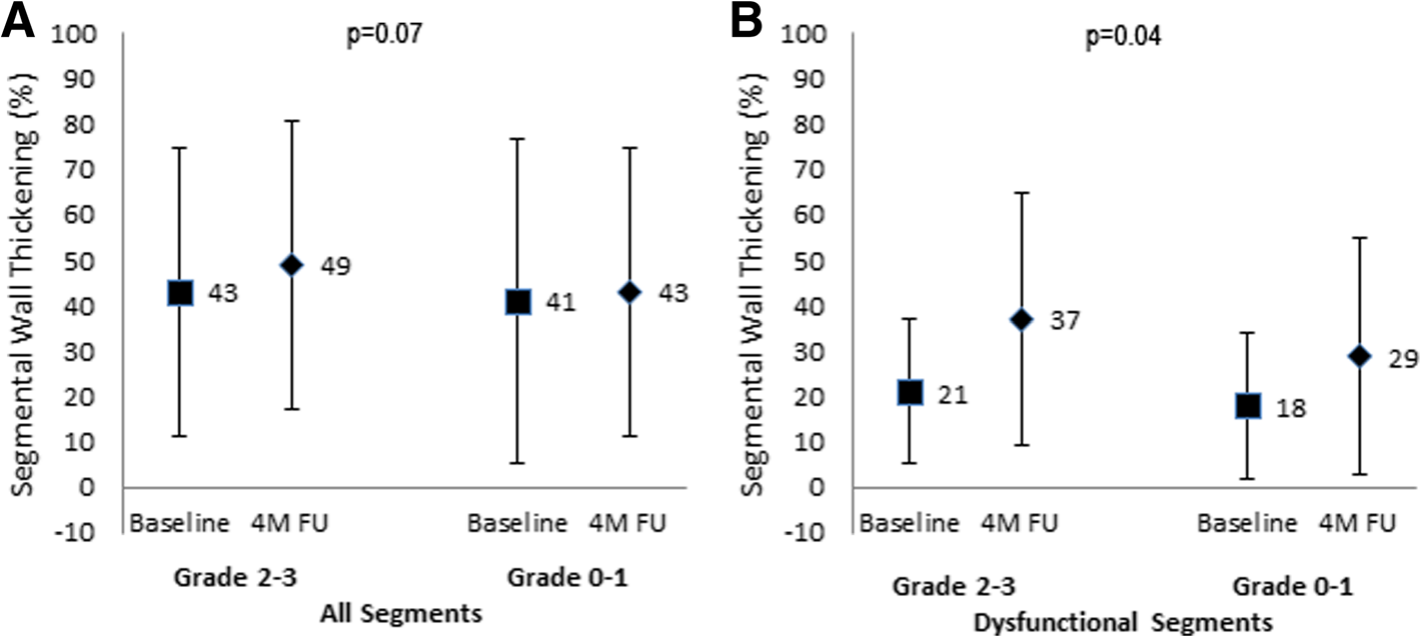
Changes in regional left ventricular function in segments in the CTO territory in Rentrop grade 2–3 collaterals versus grade 0–1 collaterals, comparing CTO PCI versus no-CTO PCI. Change in percentage segmental wall thickening (SWT) in all segments (**a**) and dysfunctional segments (**b**) in the CTO territory comparing Rentrop grade 2–3 collaterals versus grade 0–1 collaterals. Recovery of SWT was compared between baseline and 4 month follow-up

In the CTO territory the recovery of SWT of segments was compared in patients with Rentrop grade 2–3 versus grade 0–1 collaterals to the CTO territory. Baseline SWT of all the segments in the CTO territory was not different between the patients with Rentrop grade 2–3 collaterals (43 ± 32%) and the patients with grade 0–1 collaterals to the CTO (41 ± 36%) (difference in baseline SWT: p = NS). In dysfunctional segments in the CTO territory recovery of SWT was better in the patients with Rentrop grade 2–3 collaterals compared to grade 0–1 collaterals (16 ± 26% versus 11 ± 24%, *p* = 0.04) (Fig. 3). However there was no significant interaction of collateral grading on the effect of CTO PCI on recovery of SWT in the CTO territory (*p*-value for interaction *p* = 0.74).

## Discussion

### Impact of CTO PCI on regional myocardial function

This EXPLORE CMR study is the first randomized study evaluating the effect of CTO PCI on the recovery of regional LV function in STEMI patients with a concurrent CTO at 4 month FU. Furthermore this is the only study that compares an invasive treatment strategy with a control group not undergoing CTO PCI within 4 months. In addition this sub-analysis is performed in the largest cohort of serial CMR data in the CTO field. The key finding of our study is that revascularization of the dysfunctional myocardium supplied by the CTO results in a greater recovery of regional LV function from baseline to 4 months FU, when compared with no revascularization of the CTO territory.

The effect of CTO PCI on regional myocardial function is sparsely investigated. Previous smaller studies, all without a control group, have suggested an improvement of regional wall motion after successful CTO PCI, especially in initially dysfunctional segments. Furthermore, these studies showed that improvement in the CTO territory was only observed in segments without TEI (viable segments) [7]. Kirschbaum et al. also suggested that in elective CTO patients undergoing CTO PCI early and late improvement of regional LV function was related to the percentage of TEI of the CTO area [6]. Another small study showed that the extent of dysfunctional but viable myocardium at baseline was related to the improvement of SWT after CTO PCI [15]. The aforementioned studies were all done in elective and only successful CTO PCI patients. Our study confirms previous studies. Overall, the benefit of CTO PCI is most evident in initially dysfunctional but viable segments (TEI < 50%). In dysfunctional segments with a TEI >50% there was no significant difference in improvement after CTO PCI compared to no-CTO PCI, although number of segments are small. However previous studies showed that the diagnostic performance of contrast enhanced CMR is less evident in the segments with intermediate TEI [6, 15]. Additional viability testing with stress (dobutamine) perfusion CMR leads to better prediction of improvement in dysfunctional myocardium after CTO PCI than the parameter TEI alone [5]. The marked improvement of regional function in dysfunctional but viable segments, emphasizes the need for randomized controlled trials to investigate the optimal CMR assessment of dysfunctional and viable myocardium and which CMR parameters are able to accurately assess which patients will benefit from CTO PCI.

In both treatment groups there was an increase of SWT at 4 months, probably due to recovery of the stunned myocardium, caused by acute interruption of blood flow. Recovery of stunned myocardium will lead to (partial) recovery of LV function, typically over hours to days [16]. In a chronic situation recovery is related to the extent of hibernating myocardium of the targeted territory, and usually will only improve after restoration of blood flow. Hibernating myocardium is defined as myocardium that has adapted to the chronic reduction in blood flow in order to preclude actual infarction, but resulting in loss of myocardial contraction [17]. Previous studies have shown that functional recovery of hibernating myocardium can vary from 10 days, in case of minor structural abnormalities, up to 14 months [18, 19]. Therefore it is possible that, in our unique population, recovery was delayed and that the functional recovery was incomplete at 4 months. In a previous study of patients with chronic ischemic LV dysfunction there was a positive relation between the amount of dysfunctional but viable myocardium at baseline and improvement in LVEF at six months follow-up. In this study a cut-of value of ≥55% for viability was able to predict both the long-term significant improvement in LVEF and the reverse LV remodeling [20]. A previous study in patients with coronary artery disease and with wall motion abnormalities, but without infarction, showed that revascularization of the hibernating myocardium results in reverse remodeling and that the extent of this remodeling was related to the number of viable segments [21]. This reverse remodeling can lead to improvement in outcome and less future cardiac events. Therefore longer follow-up CMR studies are needed to assess the effect of regional recovery on global LV function in STEMI patients with a CTO.

Furthermore, the effect of CTO PCI on regional LV function might be associated with other positive effects of CTO PCI such as prevention of arrhythmias. Presence of a CTO affects the electrophysiological properties of the myocardium and restoration of antegrade blood flow to the ischemic and dysfunctional CTO territory might enhance electrical stability. In ICD patients with a CTO, compared to patients without a CTO, more ICD therapy was delivered [22]. This indicates that the persistent ischemic zone of the CTO territory, consisting of re-entry circuits and myocardial cells with abnormal automaticity at the border zone of previous MI, is a substrate for such arrhythmias [23]. In elective setting a significant decrease of both corrected and uncorrected QT-interval dispersion on electrocardiography was directly seen after successful CTO PCI compared to failed CTO PCI, indicating improvement of regional myocardial repolarization [24]. This indicates that the hibernating myocardium in the CTO territory might improve in repolarization duration shortly after successful CTO PCI. Therefore the effect of revascularization on regional LV recovery might also be true for restoration of repolarization duration immediately after CTO PCI while more time is needed for the recovery on a more mechanical level (contractile function). This effect on electrophysiological properties might in turn lead to a reduction in incidence of arrhythmia and sudden death, even though there is little or no effect on global functional outcome. However what the timing and the prognostic effect of this electrical recovery actually is needs further exploration.

In our study segments in the CTO territory showed better recovery of SWT in case of Rentrop grade 2–3 collaterals compared to grade 0–1 collaterals. In single CTO patients without a prior MI, patients with Rentrop grade 3 collaterals showed less resting regional wall abnormalities with minimal resting perfusion abnormalities on myocardial perfusion SPECT. However, most patients did have stress induced perfusion defects. These findings suggest that chronic stunning rather than true hibernation is the primary cause of regional wall abnormalities [25]. This stunned myocardium will recover faster than the hibernating myocardium, theoretically leading to earlier functional recovery and probably improved outcomes. In a previous study of STEMI patients with a concurrent CTO survival was better in the patients with well- versus poorly-developed collaterals [26]. However the exact role of collaterals on LV functional recovery and outcome remains controversial and results are conflicting.

### Study limitations

There are several limitations applicable to this study. Our sample size might be too small to detect differences, especially in the various subgroups. Unfortunately, baseline CMR was not performed in all patients included in the EXPLORE trial, though the baseline characteristics are comparable between patients with and those without serial CMR. Overall the patients included in the study had a moderately reduced LVEF, therefore the results might not be applicable to all patients, especially the patients with a severely reduced LVEF or patients presenting with cardiogenic shock. However SWT analysis was a predefined secondary endpoint of the EXPLORE trial.

## Conclusions

In dysfunctional segments in the CTO territory, CTO PCI compared with no-CTO PCI was associated with a marked improvement of regional function. Furthermore, in dysfunctional segments with TEI < 50% in the CTO territory CTO PCI compared to no-CTO PCI resulted in a better recovery of SWT, while in dysfunctional segments with TEI > 50% there was no treatment effect on regional function. Furthermore patients with grade 2–3 collaterals compared to grade 0–1 collaterals to the CTO showed a significantly better SWT recovery in the dysfunctional segments. Further studies are needed to investigate the effect of this regional improvement on clinical outcome and how to use CMR in selecting patients for CTO PCI after STEMI.

## Abbreviations

CMR: Cardiovascular magnetic resonance
CTO: Chronic total occlusion
EF: Ejection fraction
EXPLORE: Evaluating Xience and left ventricular function in PCI on occlusiOns afteR STEMI
ICD: Implantable cardioverter-defibrillator
IRA: Infarct related artery
LV: Left ventricular
LVEDV: Left ventricular end-diastolic volume
PCI: Percutaneous coronary intervention
STEMI: ST-segment elevation myocardial infarction
SWT: Segmental wall thickening
TEI: Transmural extent of infarction
